# Comprehensive Format of Informed Consent in Research and Practice: A Tool to uphold the Ethical and Moral Standards

**DOI:** 10.5005/jp-journals-10005-1411

**Published:** 2017-02-27

**Authors:** P Arun Bhupathi, GR Ravi

**Affiliations:** 1 Assistant Professor, Department of Orthodontics, Vishnu Dental College, Kovvada Andhra Pradesh, India; 2 Reader, Department of Pedodontics and Preventive Dentistry, Drs Sudha and Nageswara Rao Siddhartha Institute of Dental Sciences, Allapuram, Andhra Pradesh, India

**Keywords:** Ethical issues, Human research, Informed consent.

## Abstract

Informed consent in research, clinical trial, and practice is a process in which a patient/participant consents to participate or undergo the proposed procedures after being informed of its procedures, risks, and benefits. Ideally, the patient/participant is expected to give his consent only after fully comprehending the information about the procedures, benefits, and risks involved in research/clinical trial/practice. Thus, many ethical issues are entwined in the process of obtaining a proper informed consent. Certain untoward events in the past led to propose guidelines to prevent exploitations and unhealthy practices in the field of life science. Eventually, the practice of obtaining informed consent was emphasized to make sure that a participant’s rights were not in jeopardy. Yet, there are flaws in the practical application of obtaining consent due to lack of understanding, barriers in communication, culture, custom, and various other factors. The present article highlights the need for a complete and comprehensive format of recording informed consent without compromising the rights of an individual and the standards of research or practice on ethical and moral grounds.

**How to cite this article:**

Bhupathi PA, Ravi GR. Comprehensive Format of Informed Consent in Research and Practice: A Tool to uphold the Ethical and Moral Standards. Int J Clin Pediatr Dent 2017;10(1):73-81.

## INTRODUCTION

New advents in science and technology have expanded the horizons of every field including the field of medicine. Concomitantly expensive health care and scarcity of the required resources and demanding expectations of the public have led to a paradigm shift in the concepts of certain old ethical practices. Thus, new questions concerning the ethical principles are being raised time and again to adapt to the changing scenario.

Most of the biomedical research is conducted in the developing countries, which are known to have limited resources and the populations live in high-risk health conditions. Further, social and cultural factors and beliefs vary, raising the ethical concerns, such as standard of care and posttrial obligations. Henceforth, the assurance for conducting research in these countries is being discussed very often.^1^

For centuries, general medical practice has been guided by ethical principles and the basics can be dated to the Hippocratic code of conduct, which specifies that the physician will use the treatment to help the sick according to his ability and judgment, but never with the view to injury and wrongdoing. However, there was relative paucity of universally agreed guidelines or a framework for the ethical conduct of research, including medical experimentation. The Nuremberg Code of conducting research on human subjects was put forth following the atrocities post-World War II and, in 1964, the Helsinki Declaration was drafted by the World Medical Association. This was the first of its kind, a move toward developing guidelines for ethical regulation globally.^2,3^ An important component of conducting research in any setting is obtaining informed consent, as it has been the cornerstone for ethical conduct and regulation of research. It is the focus of attention in the guidelines of conducting research and the ethical oversight of research.^3^

The basic rights of a person cannot be ignored since the autonomy and responsibility of every person to decline or take part in the research is of extreme importance. The decisions concerning one’s own body or health is universally recognized as a right. Hence, emphasis is placed on the importance of informed consent in research as well as clinical practice settings, and the need of it to be enterprising and innovative in obtaining it is justified.^1^

The purpose of obtaining informed consent as a protocol for planned treatment differs from that obtained for research context. This is because level of protection for the patients varies when compared with the research subject. As the levels of protection differ, exceptions to the policy have been allowed for situations when obtaining consent is impossible or not feasible. As the consent should be suitable to varying circumstances, they may be broadly categorized into implied consent, written consent, expressed consent, informed consent, proxy consent, loco parentis, blanket consent, and oral consent.^4^

The purpose of this article is to highlight the importance of a complete, comprehensive format of consent, which upholds the rights of the individuals without compromising the standards of the research on ethical and moral grounds.

## INFORMED CONSENT

### Definition

Consent has been defined by Webster’s Dictionary as “to give assent or approval.” This statement needs to be changed when applied to various fields, dentistry being no exception. The European Commission on ethical research has defined it as “Informed Consent is the decision, which must be written, dated and signed, to take part in a clinical trial, taken freely after being duly informed of its nature, significance, implications and risks and appropriately documented, by any person capable of giving consent or, where the person is not capable of giving consent, by his or her legal representative; if the person concerned is unable to write, oral consent in the presence of at least one witness may be given in exceptional cases, as provided for in national legislation.”^5^

The British Dental Association’s “ethics in dentistry” advice sheet has defined the process of expressing consent as “A patient gives express consent when he or she indicates orally or in writing, consent to undergo examination or treatment or for personal information to be processed.”^5^

The Health Care Consent Act, 1996 Ontario has highlighted the salient features for informed consent, which include: (1) nature of proposed treatment, (2) expected benefits, (3) material risks and side effects, (4) alternative courses of action, (5) consequences of not having the proposed treatment, (6) answers to any questions the patient has regarding the proposed treatment, and (7) cost of the treatment.^6^

An informed consent form is mandatory when the research/clinical trial involves any human volunteer like children, differently-abled individuals, immigrants, or healthy individuals. It is also required whenever the personal data, biological samples or specimens, or human genetic material are used or collected.^5^

### General Format for Consent—Practice and/or Research

**Fig. 1:**
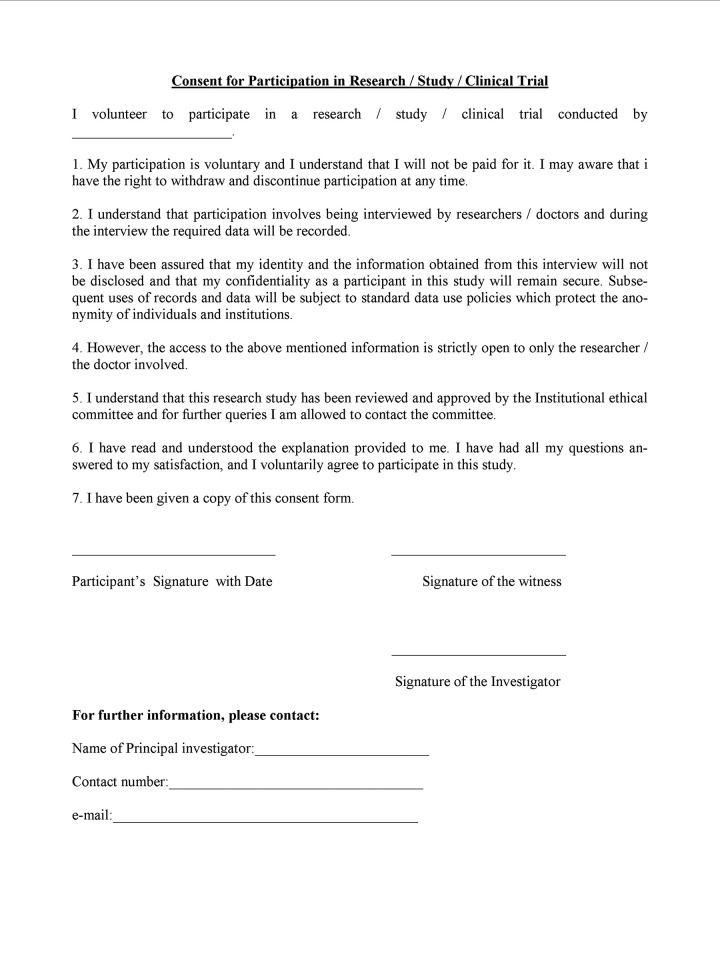
Consent form

Commonly used formats of the consent include a statement that confirms that the participant has been explained about the proposed treatment plan/clinical trial/research and his/her participation is voluntary (Fig. 1). There is a provision for the witness to sign in the document to authenticate that the above-said protocol was followed in his/her presence. In addition, there will be the details of the investigator. The informed consent is considered to be valid only when the participant, investigator, and the witness sign the document at their designated areas.

### Limitations

In this format, the content of the informed consent can be considered to be inadequate for the following reasons. This does not provide any written evidence explaining the role of the participant, investigator, and translator. Further, it lacks the structured format of explanation, which enables the participant to read about the proposed study design/treatment plan; risks involved; and assurance about the confidentiality of the identity. There is no separate declaration for participant, investigator, and translator, which commits each of them to their duties.

Thus, an informed consent that upholds the rights of the individuals without compromising the standards of the research on ethical and moral grounds is needed. This can be formulated by adapting the guidelines of the Helsinki Declaration.

### Importance of having the Consent as per Helsinki Declaration

If the informed consent is designed as per the norms of the International Declaration of Helsinki, it upholds the safety of those participating in research as well as seeking treatment in the practice. All the details shown in the template have to be filled for proper documentation. For better understanding, the entire format can be categorized into three parts. The initial part of the document should have the details of the title of the research/study along with the name, address, and contact details of the principal investigator, and the ethical committee reference number. The second part should consist of patient information sheet (Fig. 2A), and the consent certificate or the declaration should be the last part (Figs 2B to D). The entire informed consent should be printed on the letter head of the institution or the organization, which is carrying out the proposed research or clinical trial.^7,8^ The header of the document should have logo, name, and the complete postal address of the organization or the institution, which is carrying out the research/study/ clinical trial, while the footer should have the research title and date.

**Fig. 2A:**
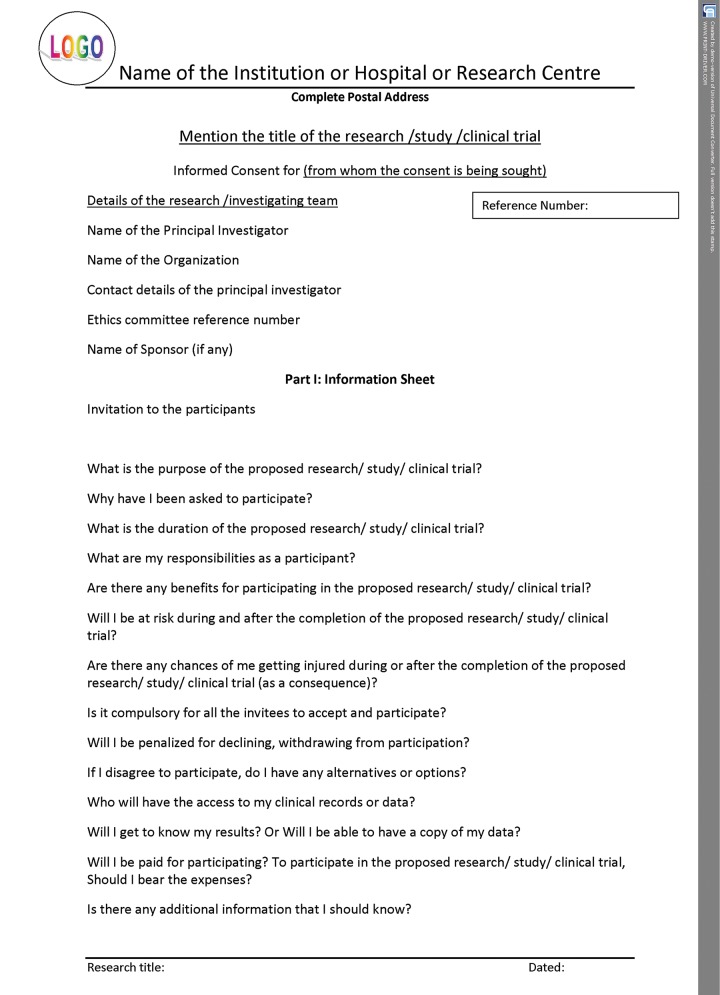
Consent form in Helsinki declaration format—patient information sheet

**Fig. 2B:**
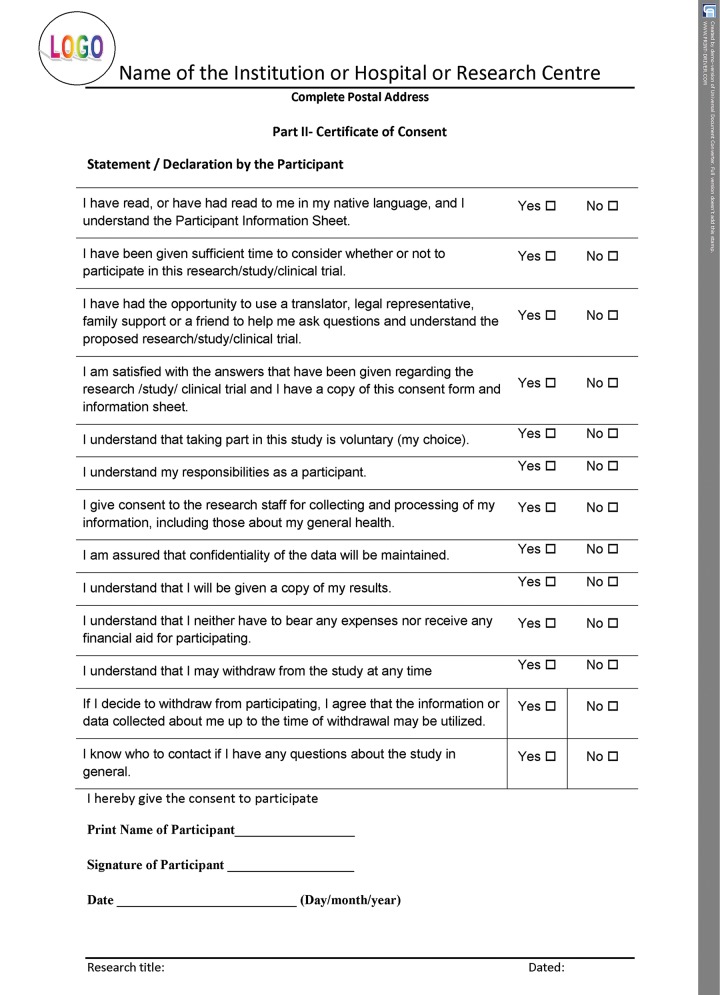
Consent form in Helsinki declaration format—statement or declaration by participant

**Fig. 2C:**
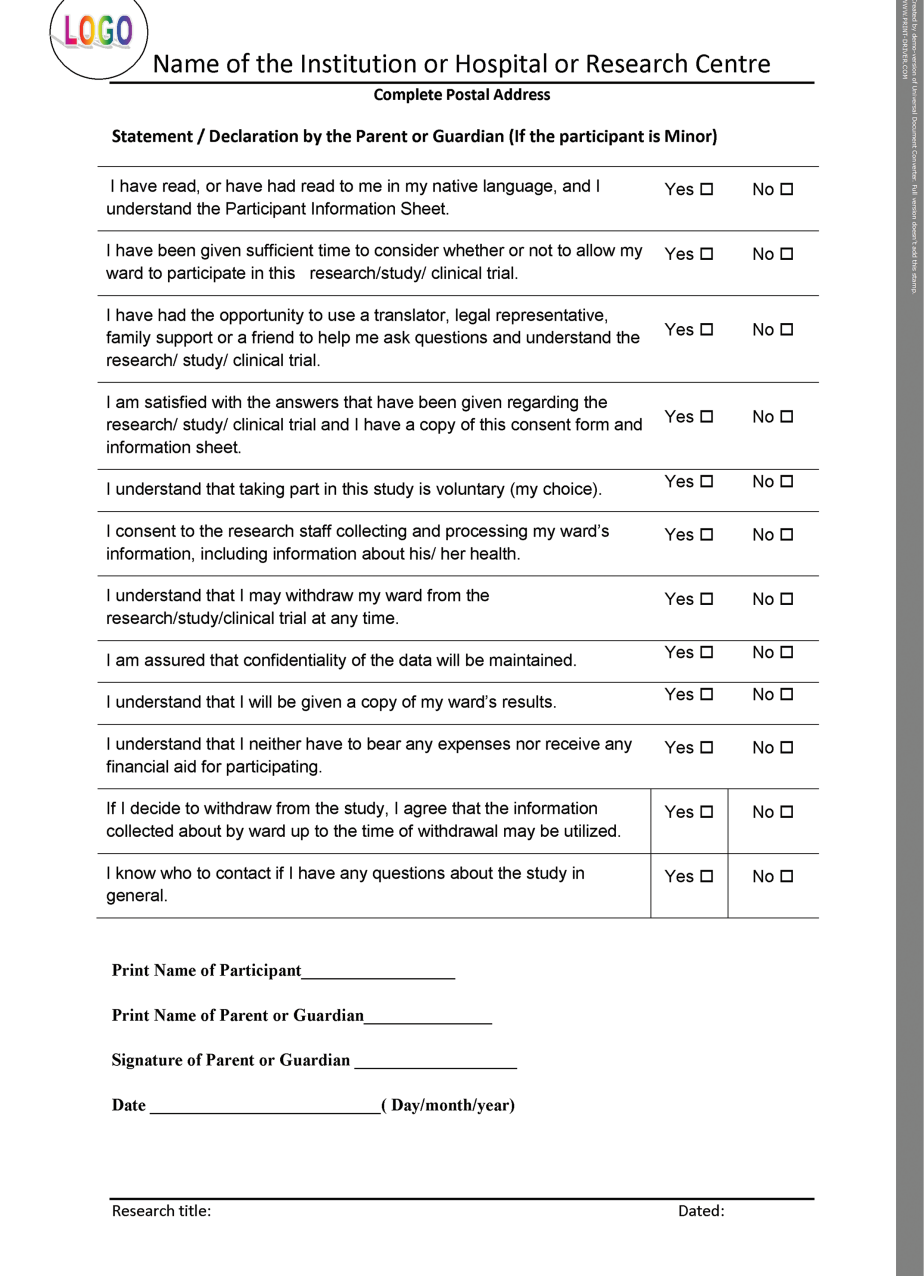
Consent form in Helsinki declaration format—statement or declaration by parent or guardian (if the participant is minor)

**Fig. 2D:**
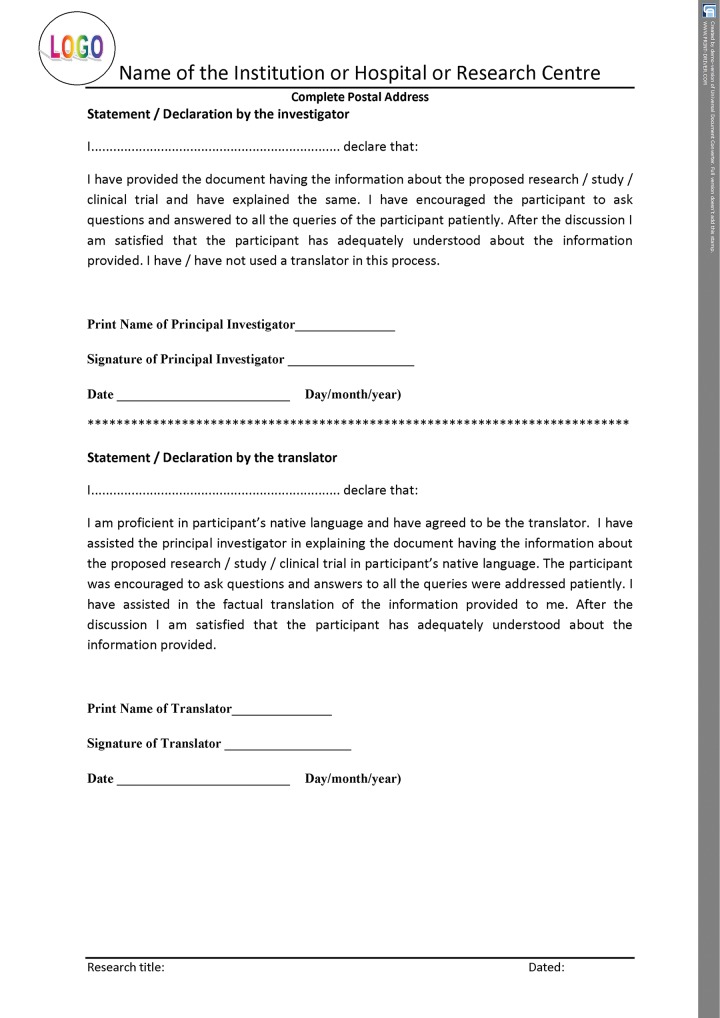
Consent form in Helsinki declaration format—statement or declaration by principal investigator and translator

## PATIENT INFORMATION SHEET

### Invitation to the Subjects to participate in the Proposed Study

The participants should be invited to take part in the proposed research/study/clinical trial. The participant must be instructed to take some time to read the information presented here, which will explain the details of this project. Then, assure them that they are free to ask the study staff/doctor/investigator any questions about any part of this project and clarify their doubts, as it is very important for them clearly understand and be fully satisfied with the details of the proposed research. This will further help the participants in knowing their involvement in the study. It should be clearly stated that their participation is “entirely voluntary” and the individual is free to decline to participate. If declined, this will not affect them by any means. It should also be mentioned that participant is free to withdraw from the study at any point, even if after agreeing to take part.

Prior approval from the Committee for Human Research/Institutional Ethical Committee of the concerned dental or medical college or the hospital has to be obtained. Further, it has to be declared that the proposed study will be conducted according to the ethical guidelines and principles of the International Declaration of Helsinki, guidelines of the statutory body involved, and the Medical Research Council—Ethical Guidelines for Research of the country.

Questionnaire-based patient information sheet is usually designed as it enables the participant to understand better. The proficiency of the language used should be simple.^8^ The description of the details to be covered in the questionnaire is explained below.

### What is the Purpose of the Proposed Research/Study/Clinical Trial?

Describe the details of the study in terms of:

- Aims and objectives of the study.
- Why this study has to be done?
- How this study is intended to be done?
- How are the observations of the study going to be useful to the individual/community?

### Why have I been asked to participate?

Inform the participant that he or she has been chosen to participate because he/she would fulfill the selection criteria. Explain briefly the aims and objectives of the studies based on which selection is made.

### What is the Duration of the Proposed Research/Study/Clinical Trial?

The duration required for the completion has to be mentioned clearly to all of the participants. This is beneficial to both the participant and the investigating team as it prevents bias due to sample attrition

### What are My Responsibilities as a Participant?

The participants should provide the required information/samples/specimens whichever is required as per the study/research/clinical trial.

### Are there any Benefits for participating in the Proposed Research/Study/Clinical Trial?

The participants have to be explained that he/she will not benefit from this research directly by themselves. Their participation would, however, be very valuable, as this contributes to medical/dental knowledge, in general. Further, it might lead to develop new diagnostic or preventive measures and better treatment modalities.

### Will I be at Risk during and after the Completion of the Proposed Research/Study/Clinical Trial?

If any risks are involved in the research, they should be clearly explained and how it could affect the individual in future.

### Are there any Chances of Me getting injured during or after the Completion of the Proposed Research/Study/Clinical Trial (As a Consequence)?

If applicable, it has to be clearly explained, as how this would affect the individual’s life. If not applicable, assurance has to be given about the same.

### Is It Compulsory for all the Invitees to accept and participate?

No, it is never a compulsion to the invitees to accept and participate. It is absolutely voluntary. Further, every individual can withdraw from participation at any given point of time.

### Will I be penalized for declining, withdrawing from Participation?

None of the invitees or participants will be penalized for declining or withdrawing from participation.

### If You disagree to participate, do I have any Alternatives or Options?

Assurance should be given to every individual who declines to participate that it is never going affect them in seeking the heath care.

### Who will have access to My Clinical Records or Data?

A statement should be issued assuring that all personal information collected will be treated as confidential and access to it will be strictly controlled and limited to the team of investigators. All identifying information will be made anonymous at the earliest possible time point and all the specimens will be designated by numbers for identification purposes when used in a publication or thesis.

### Will I get to know My Results? Or will I be able to have a Copy of My Data?

Yes, every participant is entitled to know their results and to receive a copy of it.

### Will I be paid for participating? To participate in the Proposed Research/Study/Clinical Trial, should I bear the Expenses?

It has to be clearly mentioned that no one will be paid for taking part in the study, and the participant will not incur any costs either.

### Is there any Additional Information that I should know?

Assurance should be given that:

- Participants can contact the principal investigator at the given telephone number if they have any further queries or encounter any problems.
- Participants can contact the committee for human research/institutional ethical committee at the given contact number if they have any concerns or complaints that have not been adequately addressed by their study doctor.
- Participants will receive a copy of this information and consent form for their own records.

## CONSENT CERTIFICATE AND DECLARATION

Separate declaration should be signed by the participant (Fig. 2C), investigator, and translator as shown in the template (Fig. 2D). This ensures that all the signatories are aware of their duties and are committed to the process by giving their consent.

## CONCLUSION

It has been 50 years since the International Declaration of Helsinki was drafted for protecting the human rights in the field of research. Yet, it is not being well acclaimed, especially in the developing countries. Hence, a thorough knowledge is required to understand and implement the doctrine of consent process in populations with different ethnic, race, and cultural backgrounds. This would not only uplift the ethical and moral values of the profession, but also ensures the practice of obtaining genuine and voluntary informed consent. This can be achieved by strictly adhering to the principles of the International Declaration of Helsinki as every health professional has the mission of safeguarding the health of the people.
